# Caco-2 Cell Sheet Partially Laminated with HT29-MTX Cells as a Novel In Vitro Model of Gut Epithelium Drug Permeability

**DOI:** 10.3390/pharmaceutics15092338

**Published:** 2023-09-18

**Authors:** Yi Cheng, Chie Watanabe, Yusuke Ando, Satoshi Kitaoka, Yuya Egawa, Tomoya Takashima, Akihiro Matsumoto, Masahiro Murakami

**Affiliations:** 1 Laboratory of Pharmaceutics, Faculty of Pharmacy, Osaka Ohtani University, 3-11-1 Nishikori-kita, Tondabayashi 584-0854, Osaka, Japanwatanat@josai.ac.jp (C.W.); matumoaki@osaka-ohtani.ac.jp (A.M.); 2 Laboratory of Clinical Pathology, Faculty of Pharmacy and Pharmaceutical Sciences, School of Pharmacy, Josai University, 1-1, Keyakidai, Sakado 350-0295, Saitama, Japan; 3 Laboratory of Physical Pharmacy, Faculty of Pharmacy and Pharmaceutical Sciences, School of Pharmacy, Josai University, 1-1, Keyakidai, Sakado 350-0295, Saitama, Japan

**Keywords:** Caco-2, HT29-MTX, in vitro model, intestinal permeability, partially laminated model

## Abstract

The intestinal epithelial Caco-2 cell monolayer is a well-established in vitro model useful for predicting intestinal drug absorption in humans. Coculture models of Caco-2 and goblet-cell-like HT29-MTX cells have been developed to overcome the lack of a mucus layer; however, those models are much leakier compared to the intestinal epithelium. Here, we developed a partially laminated culture model where HT29-MTX cells were superimposed onto a Caco-2 monolayer to overcome this issue. A morphological study showed that the piled HT29-MTX cells were voluntarily incorporated into the Caco-2 monolayer, and mucus production was confirmed via periodic acid-Schiff and mucin protein 2 staining. Permeability was evaluated in terms of transepithelial electrical resistance (TEER) and the apparent permeability of paracellular markers with different molecular sizes. The partially laminated model maintained the high barrier function of the Caco-2 monolayer, whose permeability appeared adjustable according to the HT29-MTX/Caco-2 cell ratio. In contrast, the coculture models showed abnormally high permeability of those markers, correlated with low TEER. Thus, the partially laminated model enabled in vitro recapitulation of effective mucosal barrier function. Consequently, this novel model may be useful as an in vitro high-throughput evaluation system for enteral mucosal permeability and mucus-penetrating efficiency of drugs and nanocarriers.

## 1. Introduction

Culture monolayers of Caco-2 intestinal epithelial cells derived from human colorectal adenocarcinoma have been used for decades as the golden standard model of the small intestinal epithelium to predict oral drug absorption in humans [1,2,3,4]. Caco-2 monolayers are a rapid, simple, reproducible, and cost-effective tool for replicating the epithelial barrier function, and they have also been used to characterize transepithelial drug transport and evaluate novel formulations. On the other hand, they have higher transepithelial electrical resistance (TEER) that may cause underestimated paracellular absorption, probably due to the lack of anatomical relevance to the intestinal epithelium composed of multiple cell populations, including enterocytes, goblet cells, Paneth cells, endocrine cells, and stem cells [5,6]. In addition, altered expression and distribution of transporters and metabolizing enzymes [7,8,9] may also cause discrepancies with data obtained in vivo. It is suggested that Caco-2 monolayer cannot predict human intestinal absorption of some hydrophilic compounds, such as ofloxacin, pregabalin, and sotalol, due to the underexpression of associated transporters [8].

The mucus layer covering the gut epithelial surface functions as a barrier to certain drugs and delivery systems, as well as microorganisms and toxins [10]. Goblet cells are the second most abundant cells in the gastrointestinal epithelium that secrete mucus to cover the mucosal surface, acting as a physical barrier based on its viscosity and mesh structure [7]. To overcome the limitations of the Caco-2 monolayer, coculture models of Caco-2 and HT29-MTX cells, which are mucus-producing goblet-like cells, have been proposed as a more physiologically relevant model [8,11,12]. The HT29-MTX cell line was adapted from parental HT29 cells, which are also intestinal epithelial cells derived from colorectal adenocarcinoma, using methotrexate to spontaneously produce mucin [13]. It has been used to clarify the role of mucus in drug transport across the intestinal mucosa. However, the drawbacks of this coculture model have also been indicated; the most critical disadvantage is leakiness. Caco-2 and HT29 coculture models exhibit a significant decrease in TEER with an increasing proportion of HT29 cells [8,14]. The permeability of compounds absorbed via passive transport is generally much higher in the coculture model than in the Caco-2 monolayer model. This is emphasized for compounds transported via the paracellular pathway, compared to lipophilic or highly permeable compounds [15,16].

For the classical method, Caco-2 cells should be cultured for approximately three weeks to form a monolayer in a confluent state. Becton Dickinson Bioscience (Franklin Lakes, NJ, USA) has developed the BIOCOAT Transwell^®^ plate to reduce the required culture time to three days and simplify the Caco-2 cell culture [17,18,19]. Yamashita et al. modified the conventional method to develop a new short-term Caco-2 monolayer culture model prepared following a three-day-culture protocol [20]. Regardless of the coculture conditions, such as the seeding ratio of Caco-2 and HT29-MTX cells, which vary among laboratories, the coculture model permeability is difficult to control accurately. Some reports indicated that the seeding ratio, as well as the culture time and medium, should affect the permeability of the coculture models [8,11]. These factors make the coculture system more complicated and less reproducible.

To address the problems of leakiness and mucus secretion, we propose an in vitro epithelial model, where mucus-secreting HT29-MTX cells are partially laminated onto a Caco-2 monolayer. In the present study, we examined the feasibility of the new partially piled model and its preparation technology and further characterized its permeability by comparing it with the conventional Caco-2 monolayer and the Caco-2 and HT29-MTX coculture model. The HT29-MTX cell attachment onto the Caco-2 cell sheet was morphologically examined via optical and confocal microscopy, and the tight junction integrity was estimated in terms of TEER. Mucus was detected using periodic acid-Schiff (PAS) staining and immunostaining of mucin 2 (MUC2) generated by goblet cells, particularly in the large intestine. The permeabilities of three culture models were compared using the apparent permeability (*P*_app_) values of water-soluble marker compounds with different molecular weights, including Lucifer yellow and fluorescein isothiocyanate-dextran (FITC-dextran 10), and a small moderate hydrophilic drug, atenolol.

Epithelial culture models are used for high-throughput assessments of drug candidate permeability and the effects of formulations or delivery systems. Thus, we modified a protocol for the short-term Caco-2 cell monolayer model to shorten the culture time to the confluency of the new model. The present study provides a new physiological model mimicking the gut mucosal barrier, which allows us to evaluate the permeability of nanoparticle delivery systems, such as lipid nanoparticles and liposomes that can pass through the mucus layer to reach the gut epithelial surface. The partially piled model and its construction technology could serve as an in vitro representation of the gut mucosal barrier.

## 2. Materials and Methods

### 2.1. Materials

Lucifer yellow, FITC-dextran, deuterium-labeled atenolol (atenolol-d7, purity ≥ 94%), and bovine serum albumin (BSA) were purchased from Sigma-Aldrich (St. Louis, MO, USA). Atenolol (purity ≥ 98%) was purchased from Combi-Blocks Inc. (San Diego, CA, USA). Ultrapure water and acetonitrile for liquid chromatography-mass spectrometry analysis (LC-MS/MS), Triton X-100, and 4% paraformaldehyde-phosphate buffered saline (PBS) solution were purchased from Fujifilm Wako Chemicals Corp. (Osaka, Japan). Caco-2 cells (ECACC No. 86010202; RCB0988) were gifted from the RIKEN BRC through the National Bio-Resource Project of the MEX, Japan, and HT29-MTX-E12 cells (ECACC No. 12040401) were purchased from KAC Co., Ltd. (Hyogo, Japan). Dulbecco’s Modified Eagle’s Medium (DMEM), nonessential amino acids, penicillin, streptomycin, PBS, and Hank’s Balanced Salt Solution (HBSS) were purchased from Nacalai Tesque Inc. (Kyoto, Japan). Fetal bovine serum (FBS), CellTracker^TM^ Green CMFDA (C7025, purity ≥ 85%), CellTracker^TM^ Orange CMRA (C34551, purity ≥ 80%), MUC2 polyclonal antibody (PA5-21329), 4’,6-diamidino-2-phenylindole (DAPI, purity ≥ 95%), and goat anti-rabbit IgG (H+L) highly cross-adsorbed secondary antibody Alexa Fluor^TM^ 488 conjugate were purchased from Thermo Fisher Scientific Inc. (Whaltham, MA, USA). All other reagents used were of guaranteed reagent grade.

### 2.2. Cell Culture

Caco-2 cells were cultured in DMEM supplemented with 10% FBS, 1% nonessential amino acids, and 1% penicillin-streptomycin. HT29-MTX cells were cultured in DMEM containing 10% FBS and 1% nonessential amino acids. Both cell lines were cultured under 5% CO_2_, 95% relative humidity at 37 °C using a cell culture incubator (MCO-18AIC, SANYO Electric Co., Ltd., Osaka, Japan), and the media were changed every 2–3 days. Caco-2 and HT29-MTX cells were used from passages 20 to 65 and 19 to 60, respectively.

In the short-term culture model, Caco-2 monolayers were prepared according to the protocol of the Corning^®^ BioCoat^TM^ Intestinal Epithelial Cell Environment Kit (Corning Inc., Corning, NY, USA) with slight modifications with reference to the procedure reported by Yamashita et al. [20]. Briefly, Caco-2 cells were seeded at a density of 1 × 10^5^ cells/insert onto 24-well Transwell^®^ inserts (0.4 μm pore size, Corning Inc.) using a basal seeding medium. After 24 h of incubation (37 °C, 5% CO_2_), the basal seeding medium was replaced with the enterocyte differential medium. After an additional 72 h of incubation, a cell monolayer was formed, and the integrity was evaluated via TEER using an epithelial volt-ohmmeter (EVOM2, World Precision Instruments Inc., Sarasota, FL, USA). In the long-term culture model, Caco-2 cells were seeded at a density of 1 × 10^4^ cells/insert onto 24-well Transwell^®^ inserts using DMEM containing 10% FBS, 1% nonessential amino acids, and 1% penicillin-streptomycin for 21 days.

**Figure 1:**
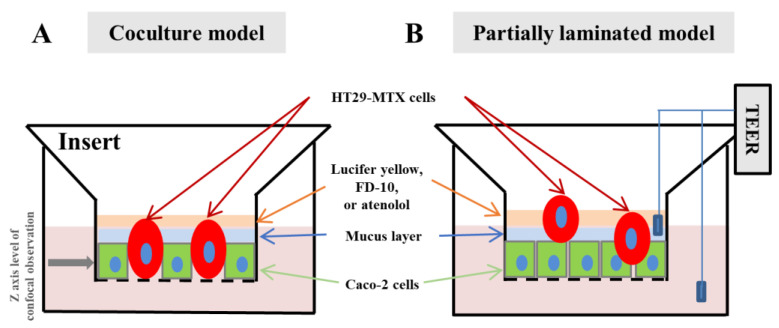
Schematic images of the partially laminated model: (**A**) Coculture model of Caco-2 and HT20-MTX cells in inserts; (**B**) Partially laminated model of Caco-2 and HT29-MTX cells in inserts; TEER, transepithelial electrical resistance.

When preparing the partially laminated model, a Caco-2 monolayer with a TEER value ≥ 2000 Ω·cm^2^ was formed first, and HT29-MTX cells were then laminated onto the Caco-2 monolayer at ratios of 1:9, 3:7, and 5:5, followed by incubation for the predetermined time. As for the coculture model, Caco-2 and HT29-MTX cells were seeded simultaneously in the different ratios and incubated for the predetermined time to form a hybrid monolayer. Those models are illustrated in Figure 1.

### 2.3. Histological Assessment

The cell sheet obtained in each culture model was histologically examined via live-cell, hematoxylin and eosin (H&E), and PAS staining. Briefly, for live cell staining, live cells were incubated with cell trackers in serum-free medium for 45 min, followed by 30 min of incubation in regular medium. Caco-2 cells were incubated with CellTracker^TM^ Green CMFDA (25 μM), and HT29-MTX cells were incubated with CellTracker^TM^ Orange CMRA (25 μM). The short-term culture models (Caco-2 monolayer, HT29-MTX monolayer, coculture model, and partially laminated model) were formed onto 24-well Transwell^®^ inserts and observed under a confocal laser scanning microscope (LSM 510 META microscope, Carl Zeiss Microscopy Ltd., Jena, Germany). CellTracker^TM^ Green CMFDA (excitation wavelength, 492 nm; emission wavelength, 517 nm) and CellTracker^TM^ Orange CMRA (excitation wavelength, 548 nm; emission wavelength, 576 nm) were observed using an excitation filter at 488 and 543 nm, respectively. Confocal images are shown as 2D (X and Y axis) images at a Z-axis sectioning surface, with white arrows indicated on the upper right corner of each image.

For H&E and PAS staining, the inserts were rinsed with prewarmed PBS and postfixed in a 4% paraformaldehyde-PBS solution overnight. After postfixation, the cell layers on the membrane in the inserts were sliced and stained by Genostaff Co. (Tokyo, Japan). The samples were observed under the bright-field view of a fluorescence microscope (BZ-X810, Keyence, Osaka, Japan).

### 2.4. Mucin Production

In addition to PAS staining, mucin formation in the cell sheet obtained in each culture model was estimated through MUC2 staining as follows: Cell layers were obtained after 3 days of incubation of HT29-MTX cells laminated onto the Caco-2 monolayer in the partially laminated model, or 4 days after Caco-2 or HT29-MTX cells were seeded for monolayer formation in the short-term culture model. Cell layers in inserts were rinsed once with 4% paraformaldehyde-PBS solution and fixed in the same solution for 15 min at room temperature. The cells were then permeabilized with 0.2% Triton X-100 for 5 min and blocked with 1% BSA for 1 h. The primary antibodies against MUC2 were added, incubated at 4 °C overnight, and rinsed three times with PBS for 5 min. The secondary goat anti-rabbit IgG antibodies conjugated with Alexa Fluor^TM^ 488 were added, incubated at room temperature in the dark for 1 h, and then rinsed three times with PBS for 5 min. For counter-staining, cells were incubated with DAPI for 5 min and rinsed with PBS. The sample was then mounted with a drop of mounting reagent, covered with a coverslip, and observed with a confocal laser scanning microscope. Alexa Fluor^TM^ 488 antibody conjugate (excitation wavelength, 499 nm; emission wavelength, 520 nm) was observed using an excitation filter at 488, and DAPI (excitation wavelength, 358 nm; emission wavelength, 453 nm) was observed using a laser 405 nm and filer BP 420–480 IR. Confocal images are shown as 2D (X and Y axis) images at the Z-axis sectioning plane, with white arrows in the upper right corner of each image.

### 2.5. TEER Measurements

The integrity of the cell sheet in each culture model was examined with culture time using TEER obtained with the epithelial volt-ohmmeter. Briefly, an insert with 400 μL medium was transferred to the EndOhm chamber containing 1 mL medium. The chamber and the cap contain a pair of concentric electrodes: a voltage-sensing silver/silver chloride pellet in the center and an annular current electrode, with the electrode position appropriately adjusted.

### 2.6. Permeability Study

The permeability of each culture membrane was determined using Lucifer yellow (MW, 521.57), FITC-dextran (average MW, 10 kDa), and atenolol (MW, 266.34) as a model drug. Briefly, the culture medium on both sides of the Transwell^®^ insert was replaced with HBSS and washed twice with prewarmed HBSS. After 30 min of incubation at 37 °C, 100 μM Lucifer yellow, 1 mg/mL FITC-dextran, or 200 μM atenolol in 200 μL HBSS were applied to the apical compartment, followed by incubation for 2 h without shaking. The amount of Lucifer yellow, FITC-dextran, or atenolol transported from the apical to the basolateral compartment was quantitatively assessed at the designated time. The *P*_app_ coefficients of the model drugs were calculated using the following equation [19,21]:

(1) $$P_{app}=\frac{dQ}{dt}\times \frac{1}{A.C_{0}}$$

where dQ/dt is the velocity of solutes transported across the culture cell sheet in time, C_0_ is the concentration of the solute in the apical compartment at time zero, and A is the cross-sectional area of the cell sheet in contact with the apical solution.

### 2.7. Analytical Methods

Lucifer yellow and FITC-dextran in the sample solution were measured via fluorescence spectrophotometry using a hybrid multimode microplate reader (Synergy H4; BioTek Instruments, Winooski, VT, USA). The excitation and emission wavelengths were 428 and 540 nm for Lucifer yellow and 485 and 512 nm for FITC-dextran, respectively.

For atenolol, 100 μL of the sample solution was well mixed with 100 μL of 10 μM atenolol-d7 dissolved in acetonitrile as an internal standard, and then centrifuged at room temperature for 10 min at 15,000 rpm. The supernatant was collected as a sample for LC-MS/MS. The analytical conditions were set as follows, referring to previous reports [22]: Measurements were carried out using a Prominence UFLC high-performance liquid chromatograph (Shimadzu, Kyoto, Japan) in combination with a 4000QTRAP (ABSCIEX, Tokyo, Japan) and an XSelect CHS C18 column (130 Å, 3.5 μm, 150 mm × 2.1 mm I.D., Waters, Tokyo, Japan) at 40 °C. The mobile phase comprised ultrapure water (10 mM ammonium formate) as A and acetonitrile as B. The flow rate was 200 μL min^−1^. The gradient conditions were 2% B to 98% B in 8 min, 98% B to 98% B in 9 min, 98% B to 2% B in 9.01 min, and 2% B to 2% B in 16 min. The injection volume was 2 μL. Detection was performed in multiple-reaction monitoring mode. For atenolol, we used 268.152 and 146.100 *m*/*z* as the precursor and product ions, respectively. For atenolol-d7, we used 275.187 and 146.100 *m*/*z* as the precursor and product ions, respectively. The collision energy was set to 37 eV for both compounds.

The standards for calibration were prepared in the range of 0.01600 μM (4.256 ng/mL) to 10.00 μM (2660 ng/mL). The calibration curve consisted of triplicate calibration standards for each concentration. The correlation coefficient (r) of the calibration curve was 0.9999. The limit of detection (3.3 σ/Slop) and the limit of quantification (10 σ/Slop) calculated based on the calibration curves were 0.02906 μM (7.731 ng/mL) and 0.08807 μM (23.43 ng/mL), respectively.

### 2.8. Statistics

Experiments were performed independently, at least in triplicate. The results are displayed as means ± standard deviation. The differences between means for two groups were statistically analyzed using Student’s *t*-test, and *p* values ≤ 0.05 were considered statistically significant.

## 3. Results

### 3.1. Effect of Caco-2/HT29-MTX Ratio on the Morphology of the Partially Laminated and Coculture Models

**Figure 2:**
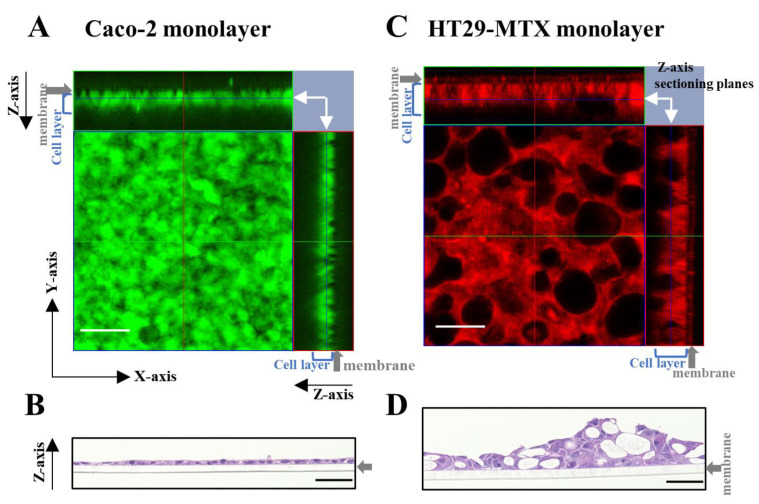
Morphology assessment of Caco-2 and HT29-MTX monocultures using live cell and hematoxylin and eosin (H&E) staining. Caco-2 and HT29-MTX cells were seeded onto the inserts and cultured for 3 days following the short-term culture protocol. (**A**) Confocal images of Caco-2 monolayer stained in green using CellTracker^TM^ Green CMFDA. (**B**) Bright-field images of the Caco-2 monolayer stained with H&E. (**C**) Confocal images of HT29-MTX monolayer stained in red color using CellTracker^TM^ Orange CMRA. (**D**) Bright-field images of the HT29-MTX monolayer stained using H&E staining. Scale bar = 50 µm. The confocal images (**A**,**C**) are shown as 2D (X-Y) images with Z-axis sectioning planes; X-Y sectioning positions are indicated with white arrows in the upper right corner.

First, we confirmed whether the Caco-2 and HT29-MTX cells were differentiated and formed a monolayer via live cell and H&E staining. In live cell staining, the presence of green color from CellTracker^TM^ Green CMFDA and red color from CellTracker^TM^ Orange CMRA indicated Caco-2 and HT29-MTX cells, respectively. As shown in Figure 2A,B, Caco-2 cells formed a compact and thin monolayer, which represented the intestinal epithelial cell layer. In contrast, HT29-MTX cells appeared to form a much looser and thicker layer, with the mucin granules clustered in the cells, showing the morphological characteristics of mature goblet cells (Figure 2C,D).

**Figure 3:**
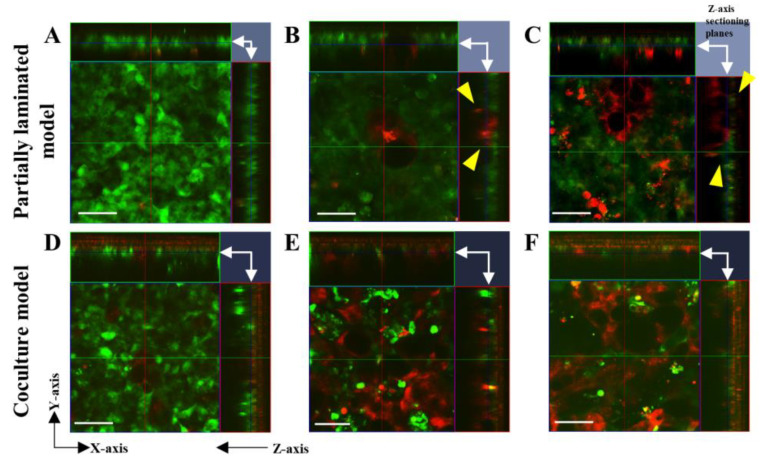
Morphology evaluation of the Caco-2/HT29-MTX partially laminated and coculture models using live cell staining. In the partially laminated model (**A**–**C**), Caco-2 cells (green) prestained with CellTracker^TM^ Green CMFDA were seeded onto the inserts and cultured for 3 days following the short-term culture protocol, and HT29-MTX cells (red) prestained with CellTracker^TM^ Orange CMRA were laminated onto the Caco-2 monolayer in ratios of 9:1 (**A**), 7:3 (**B**), and 5:5 (**C**) for another 3 days. In the coculture model (**D**–**F**), Caco-2 (green) and HT29-MTX (red) cells were mixed in ratios of 9:1 (**D**), 7:3 (**E**), and 5:5 (**F**) and cultured in inserts for another 3 days following the short-term culture protocol. The confocal images are shown as 2D (X-Y) images with Z-axis sectioning planes; X-Y sectioning positions are indicated with white arrows in the upper right corner of each image. HT29-MTX cells intercalated into the Caco-2 layer are indicated by yellow arrows (**B**,**C**). Scale bar = 50 µm.

Second, we evaluated the morphological differences between the partially laminated and coculture models. The differences between the two models were observed as the influence of HT29-MTX on cell membrane formation (Figure 3). In the partially laminated model, the green Caco-2 layer formed continuously regardless of the HT29-MTX ratio (Figure 3A–C). In addition, some HT29-MTX cells in red were intercalated into the Caco-2 layer (arrowheads of yellow triangles in Figure 3B,C). In contrast, the green Caco-2 layer was segmentalized in the coculture model with an increase in HT29-MTX, especially at an HT29-MTX ratio of 50% (Figure 3D–F). Therefore, the most significant differences in the Caco-2 layer between the two models were seen at an HT29-MTX ratio of 50% (Figure 3C,F). These results indicate that the Caco-2 layer in the partially laminated model formed firmly even under a high ratio of laminated HT29-MTX.

### 3.2. Mucus Layer in the Partially Laminated Model

**Figure 4:**
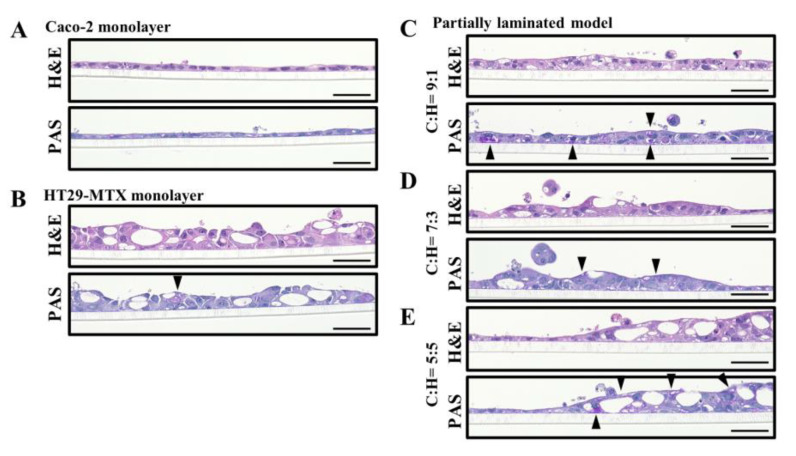
Mucus layer in the partially laminated model. H&E and periodic acid-Schiff (PAS) staining in the partially laminated model. Bright-field view of images with H&E or PAS staining of Caco-2 monolayer (**A**), HT29-MTX monolayer (**B**), and partially laminated model 3 days after the HT29-MTX cells were laminated onto the Caco-2 monolayer in Caco-2/HT29-MTX (C:H) ratios of 9:1 (**C**), 7:3 (**D**), and 5:5 (**E**). Scale bar = 50 µm.

Next, we evaluated the mucus layer in the partially laminated model, where HT29-MTX cells act as mucous-secreting goblet cells. PAS staining was performed to detect neutral mucin, glycogen production, including mucopolysaccharides, and epithelial non-sulfated simple acidic mucin. PAS and H&E staining in the continuous section showed that the layers of the partially laminated model were partially thicker than the Caco-2 monolayer (Figure 4C–E), and some areas of the cytoplasm and surface were positively stained in a strong purple color (black arrowheads in Figure 4).

**Figure 5:**
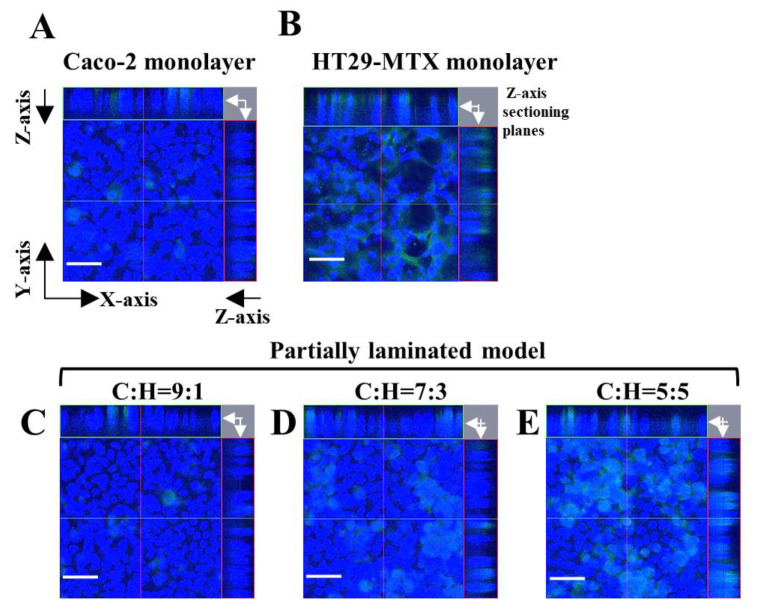
Mucin production in the partially laminated model increased along with the HT29-MTX ratio. Cells were stained with mucin 2 (green) and 4’,6-diamidino-2-phenylindole (blue) to detect mucin associated with differentiated absorptive Caco-2 cells and HT29-MTX goblet cells in the partially laminated model. Confocal images of Caco-2 monolayer (**A**), HT29-MTX monolayer (**B**), and partially laminated model 3 days after HT29-MTX cells were laminated onto the Caco-2 monolayer in C:H ratios of 9:1 (**C**), 7:3 (**D**), and 5:5 (**E**). The images are shown as 2D (X-Y) images with Z-axis sectioning planes; X-Y sectioning positions are indicated with white arrows in the upper right corner of each image. Scale bar = 50 µm.

The colon and small intestine mucus mainly comprise the gel-forming MUC2 [23]. Therefore, we examined the expression of MUC2 in the partially laminated model layers via immunostaining. The constructed cell layers were stained with anti-MUC2 primary antibodies and Alexa Fluor^TM^ 488-conjugated anti-rabbit IgG secondary antibodies. Nuclei were counterstained with DAPI, so that MUC2 was observed in green, and nuclei were observed in blue (Figure 5). MUC2 was found at the apical surface and mid-depth in the HT29-MTX monolayer (Figure 5B), but not as much in the Caco-2 monolayer (Figure 5A) and basal depth in the HT29-MTX monolayer (Figure 5B). MUC2 expression increased proportionally to the ratio of HT29-MTX in the partially laminated model (Figure 5C–E). These results suggest that laminated HT29-MTX cells may contribute to the formation of a mucin layer in the partially laminated model, which is essential to the ideal in vitro intestinal model.

### 3.3. TEER Evaluation of Partially Laminated and Coculture Model Integrities

**Figure 6:**
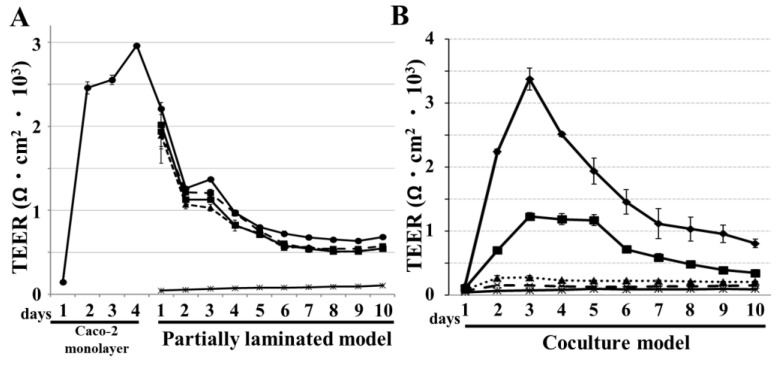
TEER variation showing integrity in the partially laminated model, but not in the coculture model. TEER values were detected in the partially laminated (**A**) and coculture (**B**) models for 10 days following the short-term culture protocol. In the partially laminated model (**A**), the Caco-2 monolayer (● with a solid line) was constructed for the first 4 days, and HT29-MTX cells were laminated onto the Caco-2 monolayer in C:H ratios of 9:1 (■ with a dashed line), 7:3 (▲with a dotted line), 5:5 (■ with a solid line), and 0:10 (✳ with a solid line). In the coculture model (**B**), Caco-2 and HT29-MTX cells were premixed in C:H ratios of 10:0 (◆ with solid line), 9:1 (■ with solid line), 7:3 (▲with a dotted line), 5:5 (✳ with a dashed line), and 0:10 (✳ with a solid line) and cocultured for the indicated days (n = 4; means ± standard deviation).

We examined the integrity of the models using TEER, which increased up to 3000 Ω·cm^2^ in the first four days when the Caco-2 monolayer was constructed via the short-term culture protocol (Figure 6A). Thereafter, it was maintained for 10 days after HT29-MTX was laminated onto the Caco-2 monolayer, then declined and reached a plateau around 500 Ω·cm^2^. TEER in the partially laminated model slightly declined in inverse proportion to the HT29-MTX cell ratio. In comparison, TEER values were extremely lower for the coculture model at 7:3 and 5:5 ratios of Caco-2/HT29-MTX cells. Even at a 9:1 ratio, the TEER values were dramatically lower than those in the Caco-2 monolayer (Figure 6B). These results indicate that the TEER of the partially laminated model was lower than that of the Caco-2 monolayer, but they could maintain a tight barrier function in the epithelial layer. In the coculture model, TEER was dramatically lower even at a 7:3 ratio of Caco-2/HT29-MTX cells.

### 3.4. Paracellular Markers Showing Permeability in the Partially Laminated and Coculture Models

**Figure 7:**
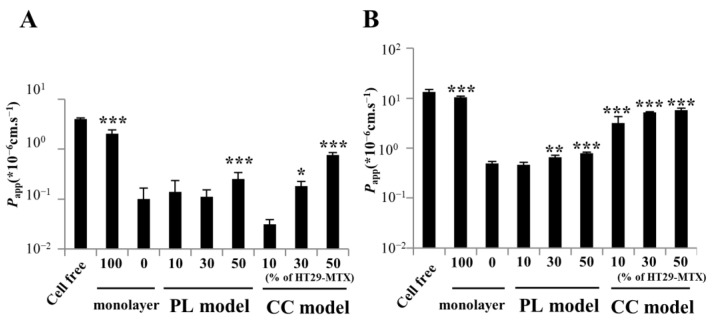
Paracellular marker permeability improved in the partially laminated (PL) and coculture (CC) models, but the latter failed to maintain the barrier function for molecules with low molecular weight. The paracellular permeability of fluorescein isothiocyanate-dextran 10 (**A**) and Lucifer yellow (**B**) was evaluated by measuring the transport from the apical to basolateral compartments for 3 h of incubation in the Caco-2 monolayer, PL, or CC models. Caco-2 monolayers served as controls (means ± standard deviation; * *p* < 0.05, ** *p* < 0.001, and *** *p* < 0.005); *P*_app_, apparent permeability.

To assess substance permeability in the partially laminated and coculture models, the *P*_app_ of FITC-dextran and Lucifer yellow was evaluated and compared to that of the Caco-2 monolayer. FITC-dextran is a fluorescent marker with high MW (average 10 kDa), and Lucifer yellow is a paracellular marker with low MW (521.57). In the partially laminated model, the *P*_app_ values of FITC-dextran at 9:1 and 7:3 ratios of Caco-2/HT29-MTX cells were similar to those in the Caco-2 monolayer, although the *P*_app_ at 5:5 ratio was >2.5 times higher than that in the Caco-2 monolayer. Moreover, the *P*_app_ of FITC-dextran in cocultures at 7:3 and 5:5 ratios was 1.8 and 7.6 times higher than that in the Caco-2 monolayer, respectively (Figure 7A). The *P*_app_ of Lucifer yellow in the partially laminated model at a 9:1 ratio was similar to that in the Caco-2 monolayer, but 1.5 times higher at ratios of 7:3 and 5:5. The *P*_app_ of Lucifer yellow in the coculture model was similar to that of the HT29-MTX monolayer, independent of the Caco-2:HT29-MTX ratio (Figure 7B). These results suggest that the coculture model shows abnormally high permeability, reflecting the leaky layer with low TEER. Additionally, we tested atenolol permeability as a paracellular marker categorized as a moderate permeability drug (50% < Fa < 84%) by the biopharmaceutics classification system [24,25]. The *P*_app_ of atenolol in the partially laminated model at a 9:1 ratio was not significantly different from that of the Caco-2 monolayer. The *P*_app_ values (×10^−3^ cm·s^−1^) of the cell-free positive control, Caco-2 monolayer, and 9:1 Caco-2/HT29-MTX partially laminated model were 14.2 ± 0.3, 0.27 ± 0.22, and 0.51 ± 0.24, respectively. Conversely, the cell layer in the partially laminated model at 9:1 or 7:3 Caco-2/HT29-MTX ratios formed tightly enough to achieve a permeability close to that of the Caco-2 monolayer, which is a more suitable in vitro intestinal cell layer model.

## 4. Discussion

Caco-2 cell monolayers have been used as a simple model for evaluating the mucosal permeability of drugs and predicting their oral bioavailability, since drugs are absorbed by the intestine mainly through the epithelial monolayer of enterocytes. On the other hand, this monolayer model has a much higher barrier function in the paracellular pathway, compared to the small intestine, and it does not have a mucus layer due to the lack of goblet cells. The latter is especially important when evaluating a colloidal drug and delivery system, because passage through the mucosal net structure is critical, and diffusion across the mucus gel layer affects the absorption rate [7].

To overcome the issues of the Caco-2 monolayer model, coculture models with mucus-secreting cells, such as HT29-MTX, have been studied in many laboratories [8,11,12,14,15,16]. However, the membranes obtained in the coculture model are so leaky that the permeability of drugs absorbed via the paracellular route can be overestimated, as mentioned in the Introduction. Therefore, to overcome the drawbacks of the coculture model, we developed a novel partially laminated model, where HT29-MTX cells are partially piled on a Caco-2 monolayer. Unexpectedly, the piled HT29-MTX cells were often intercalated into, rather than attached onto the monolayer. This backward migration-like behavior of HT29-MTX cells appears to oppose the fate of goblet cells in vivo. However, the cell layer of the partially laminated model resembled the intestinal epithelium more closely than the coculture model.

A specific junction between the two cell types should form following the invasion of HT29-MTX cells into the Caco-2 cell sheet. In vivo, immature goblet cells reside at the base of the crypt and migrate to the surface of the epithelium, differentiating to finally peel away [26,27,28]. Pearce et al. reported that adult intestinal stem cell differentiation into mature secretory and absorptive cells causes marked, but potentially reversible, changes in tight junction composition, resulting in enhanced macromolecular permeability through the junction and leading to the formation of a leak pathway between enterocytes and goblet cells [29]. Thus, although the junction composition was not investigated in the present study, HT29-MTX cell insertion into the Caco-2 monolayer may enhance junction permeability through possible composition changes. The *P_app_* values for the model compounds Lucifer yellow and FITC-dextran 10 agreed with the TEER values. In contrast, the *P*_app_ value of Lucifer yellow in the coculture model was significantly higher than that in the Caco-2 monolayer model, which suggests total barrier function loss based on the extent of compound permeability. Inadequate or poor tight junction formation is therefore possible in the coculture model. Béduneau et al. reported that 21 to 30 days, commonly used for Caco-2-based culture models, is a suitable incubation period for Caco-2 seeding to ensure practical permeability as an in vitro evaluation model [11]. In the present study, the coculture model was incubated for up to 10 days, which may be too short to complete tight junction formation between Caco-2 and goblet cells. We consider that the rapid proliferation of HT29-MTX cells may inhibit the formation of a Caco-2 membrane in this short incubation period. This may explain why the membrane obtained in the coculture model was so leaky. However, a long incubation period is inconvenient for permeability assessment in high-throughput drug screening. Therefore, a practical short-term incubation model ensuring physiological permeability is desired. The results obtained in this study suggest that the partially laminated model may provide such a practical coculture system including Caco-2 and HT29-MTX cells.

Notably, the present study demonstrated that the novel partially laminated model provides better TEER than the coculture model, including cell layers with improved permeability through the paracellular pathway. A wide range of HT29-MTX/Caco-2 cell ratios applicable for permeability assessment was obtained in the partially piled model. Most previous reports on coculture models have shown very limited HT29-MTX/Caco-2 cell ratios with practical permeability in the paracellular route [14]. In contrast, TEER in the partially laminated model was equivalent to that of the Caco-2 monolayer, and it plateaued six days after superimposition with HT29-MTX cells regardless of the ratio. This is considered important in ensuring reproducibility in high-throughput screening.

With respect to mucus production, PAS staining and MUC2 immunostaining revealed the mucus layer in the partially laminated model. The thickness of the mucus layer can affect intestinal absorption [10,16,30,31]. Mucin proteins associate with lipids, which allows for hydrophobic interactions with drugs, decreasing diffusion rates in the mucus [32,33]. Mucin oligosaccharides have a terminal carboxyl group or ester sulfate groups, which give mucus a negative net charge and the capacity to form electrostatic interactions in addition to hydrogen bonds [32]. In general, differences in mucus thickness can influence the permeability of cationic compounds and large particles. Both Lucifer yellow and FITC-dextran 10 are water-soluble anionic compounds. Diffusion of anionic particles is reportedly higher than that of neutral and cationic particles [34]. However, the influence of differences in mucus thickness is considered non-negligible, even for Lucifer yellow and FITC-dextran 10 permeability. Porcine intestinal mucus reportedly reduces the permeability of FITC-dextran 10 [35]. To control the mucus layer thickness, microbial products can increase the expression of mucins in mucus-producing cells [36,37]. Navabi et al. showed that the mucus layer in HT29-MTX-E12 cells was approximately 3–5 μm thick for 28 days of incubation post-confluency [38]. Moreover, the addition of *N*-[(3,5-difluorophenyl)acetyl]-L-alanyl-2-phenyl]glycine-1,1-dimethylethyl ester during the first six days of semi-wet interface with mechanical stimulation increased the mucus layer thickness to 25–30 μm for the 28 days of incubation. This report indicates that the incubation time and stimulation in our study were inadequate for the formation of a mucus layer. We examined mucus production in the partially laminated model using western blotting; however, glycosylated MUC2 enhancement was difficult to detect in the whole-cell lysate and supernatant, although we could observe the mucus layer via PAS staining and MUC2 immunostaining. Consequently, further studies are required to improve the partially piled model in terms of mucus layer thickness.

In a recent study, Sahoo et al. reported successful fabrication of canine-driven three-dimensional (3D) organoid monolayers and compared them using *P*_app_ with Caco-2 monolayers [39]. Human organoid technology is yet to be established due to problems, such as ethical containments and the difficulty of obtaining human biopsies. Since stable organoid passage and good reproducibility are difficult to obtain, and the medium is costly, technological progress is still required for easy and low-cost organoid production. An innovative intestinal 3D model, composed of a collagen-based stromal layer with embedded fibroblasts and epithelium prepared from Caco-2 and HT29-MTX cells cocultured for 21 days, was recently conceived [25]. This complex and advanced 3D model using Caco-2/HT29-MTX coculture can be improved by applying the partially laminated technique.

## 5. Conclusions

We developed a simple intestinal epithelial model, where HT29-MTX cells are partially laminated on a Caco-2 monolayer, resulting in a physiological structure with higher practical permeability than the conventional coculture models. It is suggested that the partially laminated model could be used to evaluate intestinal mucosal permeability of biologics and formulations, such as mucus-permeable nano-delivery systems, in a high-throughput screening process. In addition, the partially laminated technique may provide a new strategy for constructing a hybrid cell layer with different cell types in 3D models, although further intensive research is required to validate the in vitro assessment model of intestinal drug permeability.

## Data Availability

The data presented in this study are available upon written and reasonable request from the corresponding authors.
